# Genetic evaluation for production and body size traits using different animal models in purebred-Duroc pigs

**DOI:** 10.3389/fvets.2023.1274266

**Published:** 2023-12-18

**Authors:** Fuchen Zhou, Danyang Lin, Linsong Dong, Yifeng Hong, Haiyu Zeng, Gengyuan Cai, Jian Ye, Zhenfang Wu

**Affiliations:** ^1^National Engineering Research Center for Breeding Swine Industry and College of Animal Science, South China Agricultural University, Guangzhou, China; ^2^National Engineering Research Center for Breeding Swine Industry, Wens Foodstuff Group Co., Ltd., Yunfu, China

**Keywords:** Duroc pigs, common litter effect, animal model, genetic parameters, production traits, body size traits

## Abstract

Duroc pigs are popular crossbred terminal sires, and accurate assessment of genetic parameters in the population can help to rationalize breeding programmes. The principle aim of this study were to evaluate the genetic parameters of production (birth weight, BW; age at 115 kg, AGE; feed conversion ratio, FCR) and body size (body length, BL; body height, BH; front cannon circumference, FCC) traits of Duroc pigs. The second objective was to analyze the fit of different genetic assessment models. The variance components and correlations of BW (28,348 records), AGE (28,335 records), FCR (11,135 records), BL (31,544 records), BH (21,862 records), and FCC (14,684 records) traits were calculated by using DMU and AIREMLF90 from BLUPF90 package. In the common environment model, the heritability of BW, AGE, FCR, BL, BH, and FCC traits were 0.17 ± 0.014, 0.30 ± 0.019, 0.28 ± 0.024, 0.16 ± 0.013, 0.14 ± 0.017, and 0.081 ± 0.016, with common litter effect values of 0.25, 0.20, 0.18, 0.23, 0.19, and 0.16, respectively. According to the results of the Akaike information criterion (AIC) calculations, models with smaller AIC values have a better fit. We found that the common environment model with litter effects as random effects for estimating genetic parameters had a better fit. In this Model, the estimated genetic correlations between AGE with BW, FCR, BL, BH, and FCC traits were −0.28 (0.040), 0.76 (0.038), −0.71 (0.036), −0.44 (0.060), and −0.60 (0.073), respectively, with phenotypic correlations of −0.17, 0.52, −0.22, −0.13 and −0.24, respectively. In our analysis of genetic trends for six traits in the Duroc population from 2012 to 2021, we observed significant genetic trends for AGE, BL, and BH. Particularly noteworthy is the rapid decline in the genetic trend for AGE, indicating an enhancement in the pig's growth rate through selective breeding. Therefore, we believe that some challenging-to-select traits can benefit from the genetic correlations between traits. By selecting easily measurable traits, they can gain from synergistic selection effects, leading to genetic progress. Conducting population genetic parameter analysis can assist us in devising breeding strategies.

## 1 Introduction

As one of the world's most popular commercial pig breeds, the Duroc pig has undergone intense artificial selection to improve its commercial productivity and product quality (1). In recent years, Duroc pigs have gained popularity as the terminal sire in many mixed commercial lean types due to their superior performance in growth, feed conversion efficiency and conformation, along with carcass and other beneficial characteristics (2, 3). For example, Duroc × (Landrace × Yorkshire) (DLY) commercial pigs account for the majority of the current hog production market in China. During the process of breeding selection, managers preferred pigs that consume less feed, exhibit faster growth rates, possess a more robust body shape, and produce leaner meat; thereby, increasing breeding value (4). Therefore, pig breeding strategies prioritize production rates that enhance animal growth and reduce production costs (5). In this regard, birth weight (BW), age at 115 kg (AGE), and feed conversion ratio (FCR) traits are of particular interest. Among these traits, feed efficiency, which is challenging to measure directly, is often indicated indirectly by AGE (6). Moreover, body size traits such as breeding boar body length (BL), body height (BH), and front cannon circumference (FCC) play a crucial role in body size selection, whereas BL and BH are tightly connected to growth rate and physical condition (7).

Genetic improvement is a key technology for producing high-quality breeding pigs for optimal quality and sustainable production. The most valuable approach to assess the genetic worth of animals is by estimating genetic parameters such as individual breeding values (8), as they reveal the potential genetic value of traits within an animal that can be exploited through breeding and selection (5). Improving our understanding of the genetic relationships of production, growth, and body size traits in breeding pigs is necessary for updating selection indices and complementing breeding objectives (8), in addition to helping predict relevant selection responses and avoid possible undesirable side effects (9).

In contemporary breeding programs, animal models are frequently employed to calculate breeding values and variance components (10). Fixed effects, direct additive genetic effects, maternal effects, and numerous environmental effects can all be included in animal models (11). General animal, repeatability, maternal effect model, common environmental effect (litter effect), and random regression models are examples of common animal models. Choosing the most appropriate models based on the characteristics of the trait is the key to genetic parameter estimation. If some essential aspects of the assessment model are overlooked when evaluating the model, the variance may be inflated or understated (10). Thus, a well-fitting model guarantees accurate estimation of variance components, in turn producing reliable breeding value predictions. Therefore, this study analyzed the heritability, breeding values, and genetic and phenotypic correlation of BW, AGE, FCR, BL, BH, and FCC in a Duroc pig population using general animal and common environment models, respectively, to investigate (1) the influence of common environment effects on production and body size traits and (2) the genetic relationships among the traits, and (3) assess the progress of genetic improvement in this Duroc population.

## 2 Material and methods

### 2.1 Phenotypic and pedigree data sets

Phenotypic and pedigree data were collected from a great grandparent Duroc pig farm managed by WENS Foodstuff Group Co., Ltd. Phenotypic, located in Qingyuan City, Guangdong Province, People's Republic of China. The Duroc herd consists of American Duroc pigs introduced in 2010, with a current base herd size of 1,500 pigs. Phenotypic and pedigree records were collected for the period between 2012 and 2021, comprising over 130,000 phenotypic records and over 70,000 pedigree records. The study herd is distributed across six farms, with the number of individuals in each farm ranging from 144 to 24,829 pigs. The pigs were group housed in half-open cement floor pens (10 animals in each pen, with an average of 2 m^2^ per pig). Three production traits (BW, AGE, and FCR from 30 to 115 kg) and three body size traits (BL, BH, and FCC) were analyzed. Piglet birth weight was measured within 24 h after birth. The feed conversion ratio from 30 to 115 kg was assessed using an automatic feeder system called the Osborne FIRE Pig Performance Testing System (Kansas, American). Measurements were taken on the test day and adjusted to 115 kg using correction formulae derived from those independently created by WENS Foodstuff Group Co., Ltd. (formula omitted). The measurements of BL, BH, and FCC were taken when the pigs reached 115 ± 5 kg. BL was measured from the base of the ears to the base of the tail, while BH was determined from the shoulders to the ground. FCC represents the circumference of the left forelimb at its narrowest point. All three of these traits were measured using tapes. Outliers exceeding three standard deviations from the mean were eliminated prior to statistical analysis.

### 2.2 Statistical analysis

The influence of non-genetic factors, including the fixed effects of farm (six levels), year-season (42 levels), sex (two levels), and parity (11 levels), was tested for significance (*P* < 0.05) using R v4.1.2 software. Only significant effects (*P* < 0.05) were considered in subsequent mixed model analyses. DMU v6.0 (12) and AIREMLF90 program from the BLUPF90 software package were used to variance components and genetic correlation of traits using single/multiple-trait animal model. The fitness of different models was assessed using the Akaike information criterion (AIC), which is calculated as AIC = 2k - 2logL, where k represents the number of parameters used in the model and logL is the log-likelihood value estimated by AIREMLF90. The following general animal and common environment effect models were used:

Model 1: The general animal model:


$$\begin{array}{l} y\;=\;Xb\;+\;Z_{a}a\;+\;e\; \end{array}$$

Model 2: The common environment model:


$$\begin{array}{l} y\;=\;Xb\;+\;Z_{a}a\;+\;Z_{c}c\;+\;e\; \end{array}$$

where **y** is a vector of observations; **b** is a vector of fixed effects; **a** is a vector of direct additive genetic effects; **c** is a vector of common litter effects; **e** is a vector of residual effects. In this study, we define piglets born from the same mother during the same period as “common litter piglets.” Individual litter effects were recorded using the individual number of their mother along with the birth year and month as components. **X**, **Z**_**a**_, and **Z**_**c**_ are the incidence matrices for effects **b**, **a**, and **c**, respectively. It is assumed that random effects are independent and normally distributed:


$$a\;\sim \;N\;(0,A\sigma_{a}^{2}),\;c\sim \;N\;(0,\;I\sigma_{c}^{2}),\;e\;\sim \;N\;(0,\;I\sigma_{e}^{2})$$

where **A** is the numerator relationship matrix; **I** is an identity matrix; $\sigma_{a}^{2}$, $\sigma_{c}^{2}$, and $\sigma_{e}^{2}$ are the variances of random additive genetic, common litter and residual effects, respectively. Common litter effect value (***c***^**2**^) is the proportion of litter effect in the total variance, which was computed using the follow equation, $c^{2}=\frac{\sigma_{c}^{2}}{\sigma_{a}^{2}+\sigma_{e}^{2}}$.

The total phenotypic variance ($\sigma_{p}^{2}$) was calculated as $\sigma_{p}^{2}=\sigma_{a}^{2}+\sigma_{e}^{2}$, and heritability (**h**^**2**^) was computed using the following equation, $h^{2}=\frac{\sigma_{a}^{2}}{\sigma_{a}^{2}+\sigma_{e}^{2}}$ (Model 1). The total phenotypic variance ($\sigma_{p}^{2}$) was calculated as $\sigma_{p}^{2}=\sigma_{a}^{2}+\sigma_{c}^{2}+\sigma_{e}^{2}$, and heritability (**h**^**2**^) was computed using the equation, $h^{2}=\frac{\sigma_{a}^{2}}{\sigma_{a}^{2}+\sigma_{c}^{2}+\sigma_{e}^{2}}$ (Model 2).

The general animal model for two traits:


$$\left[\begin{array}{c} y_{1}\\ y_{2} \end{array}\right]=\left[\begin{array}{cc} X_{1} & 0\\ 0 & X_{2} \end{array}\right]\;\left[\begin{array}{c} b_{1}\\ b_{2} \end{array}\right]+\left[\begin{array}{cc} Z_{1} & 0\\ 0 & Z_{2} \end{array}\right]\left[\begin{array}{c} a_{1}\\ a_{2} \end{array}\right]+\left[\begin{array}{c} e_{1}\\ e_{2} \end{array}\right]$$

All elements are consistent with the single-trait model described above. The formulae for calculating the genetic correlation (**r**_**g**_) and phenotypic correlation (**r**_**p**_) between two traits were estimated according to the method of Alam et al. (5), with the genetic correlation calculated as $r_{g}=\frac{\sigma_{a1a2}}{\sqrt{\sigma_{a1}^{2}\times \sigma_{a2}^{2}}}$, where the $\sigma_{a1}^{2}$ and $\sigma_{a2\;}^{2}$ parameters are genetic variance estimates of traits 1 and 2, respectively, and **σ**_**a1a2**_ is the genetic covariance between two traits. The formula for the phenotypic correlation between two traits is denoted as $r_{p}=\frac{cov_{p1p2}}{\sqrt{\sigma_{p1}^{2}\times \sigma_{p2}^{2}}}$, where *cov*_*p*1*p*2_ represents the phenotypic covariance between the traits, and $\sigma_{p1}^{2}$ and $\sigma_{p2}^{2}$ denote the phenotypic variances of trait 1 and trait 2, respectively. In addition, we calculated breeding values for various traits of the Duroc population using the DMU v6.0 software's DMU4 module and visualized the trends in mean breeding values for all traits across birth years.

## 3 Results

### 3.1 Descriptive statistics

Table 1 presents a descriptive summary of the BW, AGE, FCR, BL, BH, and FCC traits in the Duroc population. The average values for these traits in the Duroc pigs were as follows: BW (1.66 ± 0.27 kg), AGE (185.40 ± 15.00 days), FCR (2.33 ± 0.24), BL (116.36 ± 8.30 cm), BH (62.11 ± 3.38 cm), and FCC (18.86 ± 1.09 cm). All traits had a substantial number of phenotypic records, with over 10,000 records available. Notably, the BW trait had collected 28,348 records, but also exhibited the largest coefficient of variation of 16.27%. The phenotypic coefficients of variation for the other traits ranged from 5.44 to 10.30%, indicating relatively smaller variations.

**Table 1:** Summary statistics of above six traits.

**Trait^a^**	**Number**	**Mean**	**Standard deviation**	**Range**	**Coefficient of variation (%)**
BW/kg	28,348	1.66	0.27	0.90–2.42	16.27
AGE/day	28,335	185.40	15.00	140.63–230.27	8.09
FCR	11,135	2.33	0.24	1.61–3.05	10.30
BL/cm	31,544	116.36	8.30	92.00–140.00	7.13
BH/cm	21,862	62.11	3.38	52.00–72.00	5.44
FCC/cm	14,684	18.86	1.09	16.00–22.00	5.78

^a^BW, birth weight; AGE, day to 115 kg; FCR, feed conversion ratio; BL, body length; BH, body height; FCC, front cannon circumference.

### 3.2 Fixed effects analysis

Table 2 indicates that the effects of year-season, sex, farm, and parity on traits, including BW, AGE, BL, BH, and FCC were highly significant (*P* < 0.05) and thus, were incorporated into the genetic variance statistical model.

**Table 2:** Significance test of fixed effects.

**Trait^a^**	**Year season**		**Sex**		**Farm**		**Parity**	
	**df**	**F^a^**	**df**	**F**	**df**	**F**	**df**	**F**
BW	41	24.03^***^	1	132.81^***^	5	13.04^***^	10	86.69^***^
AGE	41	237.09^***^	1	9623.12^***^	5	734.08^***^	10	18.76^***^
FCR	34	64.92^***^	1	2156.62^***^	3	0.30	9	2.48^***^
BL	41	684.55^***^	1	1791.92^***^	5	703.15^***^	10	59.83^***^
BH	34	298.50^***^	1	2578.90^***^	3	660.20^***^	8	18.40^***^
FCC	22	439.56^***^	1	5585.23^***^	2	75.78^***^	8	12.01^***^

^a^In the test of significant difference analysis of fixed effect, ^***^Indicates that the difference is extremely significant; df, degree of freedom; F, F-value.

### 3.3 Variance components and genetic parameter estimates

When Model 1 was applied in the genetic estimation model in this study, the heritability estimates for BW, AGE, FCR, BL, BH, and FCC were 0.34 ± 0.015, 0.45 ± 0.016, 0.36 ± 0.024, 0.29 ± 0.016, 0.30 ± 0.018, and 0.21 ± 0.020, respectively (Table 3), all of which were of medium heritability. However, when the common litter effect was included as a random effect in the genetic assessment model (Model 2), the estimated heritability for BW, AGE, FCR, BL, BH, and FCC decreased to 0.17 ± 0.014, 0.30 ± 0.019, 0.28 ± 0.024, 0.16 ± 0.013, 0.14 ± 0.017, and 0.081 ± 0.016, respectively (Table 4). Besides that, BW, AGE, FCR, BL, BH, and FCC exhibited common litter effect values of 0.25, 0.20, 0.18, 0.23, 0.19, and 0.16, respectively. Notably, the common litter effect values for body size traits were all higher than the heritability, indicating a large influence of the common litter effect on both production and body size traits.

**Table 3:** Variance components and heritability of each trait for Model 1.

**Trait**	** $\sigma_{\text{a}}^{2}$ **	** $\sigma_{\text{e}}^{2}$ **	** $\sigma_{\text{p}}^{2}$ **	**h^2^ ±SE**	**AIC**
BW	0.023^a^	0.045	0.068	0.34 ± 0.015	3193.7691
AGE	57.20	70.93	128.13	0.45 ± 0.016	211370.88
FCR	0.016	0.028	0.044	0.36 ± 0.024	−4491.3515
BL	2.94	7.04	9.98	0.29 ± 0.016	156708.21
BH	1.01	2.33	3.34	0.30 ± 0.018	84141.801
FCC	0.12	0.47	0.59	0.21 ± 0.020	33017.213

${\;}^{\text{a}}\sigma_{a}^{2}$, additive variance; $\sigma_{e}^{2}$, Residual errors variance; $\sigma_{p}^{2}$, phenotypic variance; h^2^, heritability; SE, standard error; AIC, the Akaike information criterion (AIC) of the model.

**Table 4:** Variance components and heritability of six traits for Model 2.

**Trait**	** $\sigma_{\text{a}}^{2}$ **	** $\sigma_{\text{c}}^{2}$ **	** $\sigma_{\text{e}}^{2}$ **	** $\sigma_{\text{p}}^{2}$ **	**h^2^ ±SE**	**c^2^**	**AIC**
BW	0.011^a^	0.016	0.038	0.065	0.17 ± 0.014	0.25	1368.5132
AGE	36.38	23.94	61.56	121.88	0.30 ± 0.019	0.20	210186.08
FCR	0.012	0.079	0.023	0.0429	0.28 ± 0.024	0.18	−3756.4275
BL	1.55	2.18	5.93	9.66	0.16 ± 0.013	0.23	155367.02
BH	0.45	0.59	2.14	3.18	0.14 ± 0.017	0.19	83430.113
FCC	0.046	0.094	0.43	0.57	0.081 ± 0.016	0.16	32597.705

${\;}^{\text{a}}\sigma_{a}^{2}$, additive variance; $\sigma_{c}^{2}$, litter effect variance; $\sigma_{e}^{2}$, Residual errors variance; $\sigma_{p}^{2}$, phenotypic variance; h^2^, heritability; SE, standard error; c^2^, the proportion of litter effect in the total variance; AIC, the Akaike information criterion (AIC) of the model.

### 3.4 Genetic and phenotypic correlation among traits

The genetic correlations (Model 1) between AGE with BW, FCR, BL, BH, and FCC traits were −0.22, 0.62, −0.54, −0.28, and −0.31, respectively, while the phenotypic correlations were −0.17, 0.49, −0.41, −0.28 and −0.23, respectively (Table 5). The genetic and phenotypic correlations revealed negative associations between AGE and body size traits, while AGE and FCR showed highly significantly positive correlations. Additionally, BW exhibited negative correlation with production traits such as AGE and FCR, and positive correlations with body size traits including BL, BH, and FCC.

**Table 5:** Genetic (below diagonal) and phenotypic (above diagonal) correlations among traits for Model 1.

	**BW**	**AGE**	**FCR**	**BL**	**BH**	**FCC**
BW		−0.17	−0.070	0.080	0.060	0.038
AGE	−0.22 (0.034)^a^		0.49	−0.41	−0.28	−0.23
FCR	−0.16 (0.051)	0.62 (0.033)		−0.25	−0.22	−0.20
BL	0.20 (0.040)	−0.54 (0.029)	−0.40 (0.047)		0.63	0.48
BH	0.13 (0.044)	−0.28 (0.038)	−0.35 (0.057)	0.75 (0.023)		0.36
FCC	0.030 (0.055)	−0.31 (0.049)	−0.48 (0.067)	0.62 (0.040)	0.51 (0.050)	

^a^The standard error of genetic correlation is in parentheses.

However, we found that the estimated genetic correlations (Model 2) between AGE with BW, FCR, BL, BH, and FCC were −0.28, 0.76, −0.71, −0.44, and −0.60, respectively, while the phenotypic correlations were −0.17, 0.52, −0.22, −0.13, and −0.24, respectively (Table 6). Similar to Model 1, AGE and FCR traits showed negative correlations with body size traits, while AGE and FCR traits exhibited significant positive correlations. Furthermore, strong positive correlations were observed among all body size traits.

**Table 6:** Genetic (below diagonal) and phenotypic (above diagonal) correlations among traits for Model 2.

	**BW**	**AGE**	**FCR**	**BL**	**BH**	**FCC**
BW		−0.17	−0.057	0.082	0.061	0.042
AGE	−0.28 (0.040)^a^		0.52	−0.22	−0.13	−0.24
FCR	−0.19 (0.059)	0.76 (0.038)		−0.25	−0.23	−0.40
BL	0.28 (0.050)	−0.71 (0.036)	−0.53 (0.061)		0.66	0.50
BH	0.18 (0.060)	−0.44 (0.060)	−0.40 (0.081)	0.84 (0.030)		0.35
FCC	0.047 (0.084)	−0.60 (0.073)	−0.48 (0.067)	0.71 (0.058)	0.56 (0.088)	

^a^The standard error of genetic correlation is in parentheses.

### 3.5 Genetic trends of production and body size traits

**Figure 1:**
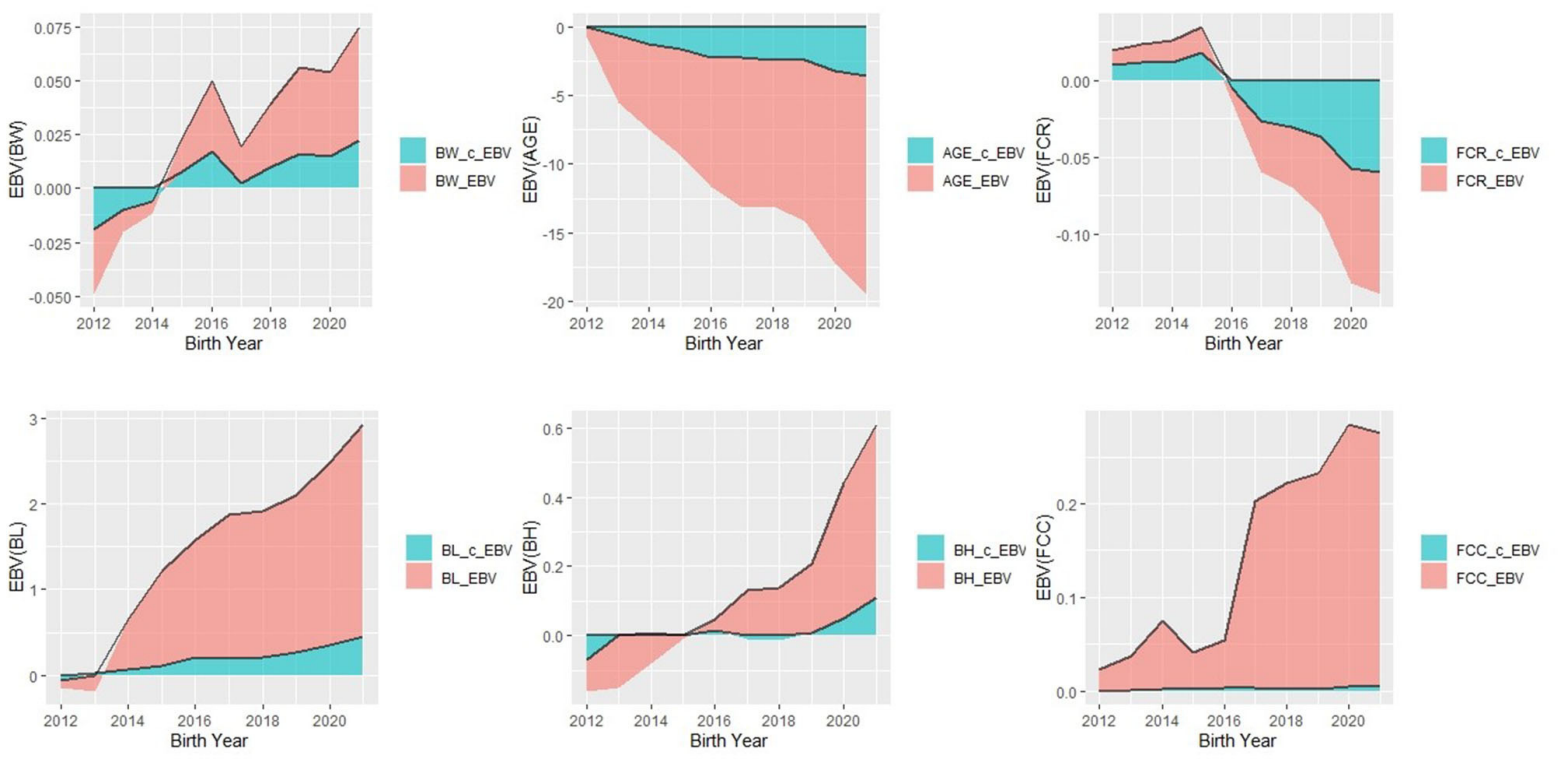
Trend of estimated breeding value (EBV) of all six traits in Duroc pigs by year of births analyzed by Model 1 and Model 2.

Figure 1 presents a comparison of the annual mean EBV for productivity and body size traits in the Duroc population estimated using models 1 and 2. Starting from 2012, the EBV for AGE traits showed a consistent decline, decreasing from −0.75 to −15.9 in Model 1 and from −0.009 to −3.55 in Model 2. Additionally, regarding the selection process for this trait, the EBV for BL traits exhibited a notable increase, ranging from −0.09 to 2.48 in Model 1 and from −0.006 to 0.45 in Model 2. Similarly, there was a consistent upward trend in BH_EBV, particularly since 2019, with values ranging from −0.09 to 0.5. However, the genetic responses for the remaining three traits displayed overall consistency between the two models, without apparent visual trends. Notably, Model 1 exhibited more pronounced predicted trends compared to Model 2.

## 4 Discussion

### 4.1 Heritability of production traits

In this study, Model 1 estimated the heritability of BW, AGE, and FCR as 0.34 ± 0.015, 0.45 ± 0.016, and 0.36 ± 0.024, respectively, while in Model 2, these values were 0.17 ± 0.014, 0.30 ± 0.019, and 0.28 ± 0.024, respectively. Furthermore, we observed that the common litter effect values of BW, AGE, and FCR were 0.25, 0.20, and 0.18, respectively. Model 2 exhibited smaller AIC values for BW and AGE compared to Model 1, while for FCR, Model 2 yielded a higher AIC. Thu s, our findings suggest that (1) the common litter effects strongly influence the aforementioned traits, and (2) Model 2 provides a better fit for analyzing genetic parameters in BW and AGE. It is worth noting that previous studies have reported a range of heritability for BW, from 0.04 to 0.44 (13–20). Notably, the finding by Jiao et al. (21) align with our study. Similarly, the heritability estimates for AGE have been reported to range from 0.25 to 0.52 (22–25). For FCR, the heritability range is reported to be 0.20 to 0.42 (16, 22, 26–29). Although our study's results are consistent with previous reports, some discrepancies exist, which may be attributed to variations in species, sample size, analytical models, and population structure. Therefore, we conclude that the heritability of the production traits is moderate to low, and selecting solely based on phenotypic measurements results in slow genetic progress. However, integrating genomic selection techniques has the potential to greatly enhance the breeding progress.

### 4.2 Heritability of body size traits

We analyzed the heritability of BL, BH, and FCC using Model 1, which were estimated as 0.29 ± 0.016, 0.30 ± 0.018, and 0.21 ± 0.020, respectively. These estimates were higher than those reported by Ogawa et al. (30) for BH but lower than their BL and FCC values. Model 2 analysis revealed heritability estimates of 0.16 ± 0.013, 0.14 ± 0.017, and 0.081 ± 0.016 for BL, BH, and FCC, respectively. These estimates were higher than those identified by Hong et al. (7) for BH but lower than their BL findings. Additionally, previous studies by Johnson and Nugent 3rd (31) and Ohnishi and Satoh (32) reported a heritability range of 0.16 to 0.38 for BL. The common litter effect values for BL, BH, and FCC were 0.23, 0.19, and 0.16, respectively. Notably, the common litter effect values exceeded the heritability estimates, indicating their significant influence on body size traits. Furthermore, Model 2 consistently yielded lower AIC values for body size traits compared to Model 1, similar to BW and AGE. Thus, we found that incorporating the common litter effect as a random factor improves the fitting of the body size trait estimation model. However, an important challenge in practical production is the precise calculation of the common litter effect. Given the disparity between postpartum sow nurturing capability and piglet physical conditions, piglet fostering is a common practice in production. Consequently, full-sib piglets may exhibit varying within-litter effects due to fostering, which cannot be determined solely by pedigree information. Therefore, we recommend that foster care should be avoided in production as much as possible. In addition to the common litter effects, factors such as maternal effects and gene-environment interactions can also enhance the accuracy of genetic evaluation for these traits. However, further research is needed to provide more support for this perspective. We consider the body size traits to have low heritability, making it challenging to achieve satisfactory genetic progress through direct selection based on phenotypic measurements. Nonetheless, by integrating genomic selection techniques, there is a great potential to significantly enhance the breeding progress.

### 4.3 Genetic correlations and phenotypic correlations

The findings of this study indicated that the BW exhibits a negative genetic and phenotypic correlation with AGE and FCR. In contrast, it shows a positive correlation with BL and BH, and a slight correlation with FCC. Furthermore, the genetic and phenotypic correlations between AGE and FCR, as determined by models 1 and 2, were found to be 0.62 and 0.49, 0.76 and 0.52, respectively. These correlations were higher than the results reported by Cheng et al. (22), providing further support for the utilization of AGE traits as auxiliary indicators in estimating FCR across different countries. Conversely, AGE was strongly negatively correlated with body size traits, such as BL, BH, and FCC. The genetic correlation between AGE and BL was observed to be −0.54 (Model 1) and −0.71 (Model 2), consistent with the findings of Nikkilä et al. (33). Additionally, this study reveals highly significant positive genetic and phenotypic correlations among body size traits, aligning with the results reported by Hong et al. (7), Zhang et al. (34), and Liu et al. (35). Therefore, in the breeding process, the incorporation of simple and cost-effective auxiliary traits such as BL, BH, and AGE can effectively aid in the selection of FCR. This approach has the potential to significantly accelerate the genetic improvement of FCR in breeding companies. Moreover, elucidating the genetic correlations among traits is essential for breeders to make necessary adjustments and specifications in breeding programs, strategically plan breeding goals, and mitigate potential antagonistic effects among traits.

## 5 Conclusion

In conclusion, both production and body size traits exhibited moderate to low heritability in this study. It is crucial to consider the influence of common litter on these traits in genetic parameter analysis. The incorporation of a common litter effect model improved the model fit and enhanced the accuracy of breeding value estimation. Furthermore, our analysis of genetic and phenotypic correlations revealed significant positive associations between FCR and AGE traits. Conversely, FCR displayed a strong negative correlation with body size traits. These findings support the use of AGE traits as auxiliary indicators for selecting FCR and suggest that pigs with larger bodies exhibit higher feeding efficiency under equivalent feeding conditions. Additionally, this study demonstrates that selecting for the BL trait alone may lead to coordinated selection of other body size traits due to strong positive genetic correlations.

## Data availability statement

The raw data supporting the conclusions of this article will be made available by the authors, without undue reservation.

## Ethics statement

The animal study was approved by South China Agricultural University. The study was conducted in accordance with the local legislation and institutional requirements.

## Author contributions

FZ: Validation, Visualization, Writing – original draft. DL: Validation, Writing – original draft. LD: Writing – review & editing. YH: Formal analysis, Writing – review & editing. HZ: Investigation, Resources, Writing – review & editing. GC: Funding acquisition, Supervision, Writing – review & editing. JY: Methodology, Project administration, Writing – review & editing. ZW: Conceptualization, Writing – review & editing.
